# Mortality in New York in 1839*From Dr. Walters’ Report of Interments.

**Published:** 1840

**Authors:** 


*Mortality in New York,* 1839.*—The whole number of interments within the city during the year ending December 31st, 1839, were 7953; being 100 less than for the year preceding.

Of these, 7491 were from among the white population, and 462 were colored persons.

Of the whole number 4389 were males, and 3564 females. This excess of mortality among the male population, as already stated in the reports of interments for previous years, is not easily explained, and is probably much greater than the proportion of male over female residents.

The disparity commences during fœtal existence, as is shown in the table of still-born infants, and continues almost through every period of life.

The average mortality among the foreign population appears to be much greater than among the native citizens. Of the whole number of deaths in persons over ten years of age, 1419 were natives and 1853 were Europeans.

The season of the year most fatal to human life in this city, as shown by the tables, is during the months of July, August

*From Dr. Walters' Report of Interments.

and September. The season, on the contrary, in which the fewest deaths occur, is during the three months immediately preceding these, namely, April, May and June.

The great mortality in this city among children under five years of age, has long been the subject of remark. During the past year, excluding the still-born, more than half the deaths, or 3696, occurred in children before the completion of their fifth year. The diseases most fatal within this period of existence are, marasmus, inflammation of the brain, hooping cough, measles, scarlet fever, dysentery, diarrhœa, cholera infantum, croup, convulsions, dropsy of the brain, and teething.

The mortality from pulmonary diseases, including in this list all the disorders of the respiratory organs, is nearly equal to one-third of the whole number of interments.

The deaths from pulmonary consumption alone, during the past year, were 1315, being an increase of 90 over the year preceding.

The mortality from pulmonary consumption in this city, may be rated at one-sixth of all the deaths; but the average varies greatly among the different classes. It is worthy of remark, that of those over ten years of age, that die of this disease, more than one-half are natives of Europe. Of the 5564 deaths among the native white citizens, only 610, or about one in nine, occurred from consumption. Of the 462 deaths among the colored population, 132, or one in three and a half, occurred from this disease. And of the 1853 deaths among the European population, 563, or about one in three and a quarter, occurred from the same disease.

The city has been visited by no fatal epidemic during the past year; and, with the exception of measles, the various contagious diseases have been less prevalent than formerly.

The tables show 68 deaths from small pox. The proportion of these that had previously undergone vaccination cannot be ascertained; but as 38 of them, or more than one-half were among children under five years of age, the probability is, that very few of the whole number had resorted to the only efficient means of protection against this loathsome and fatal malady.

*American Medical Intelligencer, May 1, 1840.*

